# Organizing Pneumonia and Ulcerative Colitis: A Relationship To Remember

**DOI:** 10.7759/cureus.36396

**Published:** 2023-03-20

**Authors:** Sergio Pereira, David Sousa, Ana Luísa Esteves, Mariana Constante, Rita Reis

**Affiliations:** 1 Internal Medicine Department, Egas Moniz Hospital, Lisbon, PRT

**Keywords:** air leak syndrome, organizing pneumonia, extraintestinal manifestation, ulcerative colitis, inflammatory bowel disease

## Abstract

Ulcerative colitis (UC) is a chronic inflammatory bowel disease characterised by relapsing and remitting mucosal inflammation of the colon. Despite primarily affecting the gastrointestinal tract, UC has various extraintestinal manifestations that often affect other organs and systems. Although pulmonary involvement is uncommon, organising pneumonia (OP), which affects the lung parenchyma, is one of the potential extraintestinal manifestations of UC. We report a case of OP in a 35-year-old male with a longstanding history of UC, well-medicated with sulfasalazine (SSZ). He presented to the emergency department (ED) with complaints of fatigue, coughing, myalgia, thoracalgia and dyspnoea. A chest X-ray showed parenchymal infiltrates and computed tomography revealed bilateral consolidation. Under a preliminary diagnosis of atypical pneumonia, he was treated with an empirical broad-spectrum antimicrobial agent, which did not lead to any clinical, laboratory or imaging improvement. Furthermore, the diagnostic work-up excluded any malignancy or infectious cause. A probable diagnostic hypothesis was OP as an extraintestinal manifestation of UC or as an adverse effect of SSZ therapy. Hence, SSZ was discontinued, and he was successfully treated with corticosteroids, exhibiting significant improvements and recovering completely during the follow-up period. Despite lung involvement in UC being rare, we present this case to emphasise the importance of a thorough differential diagnosis when treating acute respiratory illness in patients with UC, including extraintestinal manifestations of UC, especially OP, even during a period of remission. We also emphasise the importance of early initiation of corticosteroid therapy to prevent major complications and promote recovery.

## Introduction

Ulcerative colitis (UC) is a chronic, immune-mediated inflammatory bowel disease characterised by continuous inflammation of the mucosal layer, which starts in the rectum and extends to the colon [1,2]. It is clinically distinguished by periods of remission alternating with periods of exacerbation, with the typical features of diarrhoea (with or without haematochezia) that may be accompanied by abdominal pain and fever [1].

Although UC typically affects the gastrointestinal tract, it is a systemic disease that can affect other organs. These are known as extraintestinal manifestations. The musculoskeletal, dermatological, ocular, and hepatobiliary systems are the most frequently affected, while pulmonary involvement is uncommon [3,4]. The exact pathophysiological mechanisms underlying extraintestinal manifestations are still unknown [1,3].

## Case presentation

A 35-year-old male with a medical history of UC, primary sclerosing cholangitis, and autoimmune diabetes usually medicated with sulfasalazine (SSZ), budesonide, ursodeoxycholic acid, and basal-bolus insulin regimen, presented to the ED with complaints of a week-long history of fatigue, coughing, myalgia, thoracalgia, and dyspnoea. He was a non-smoker and non-drinker and had no known drug allergies. He had no history of respiratory diseases, occupational exposures, or any recent travel. He reported no recent UC flare-up episodes; his previous colonoscopy done five months before revealed no signs of active inflammation. 

He was diagnosed with community-acquired pneumonia and was discharged with oral cefuroxime. Notwithstanding the administration of oral cefuroxime, the patient's condition persisted without any improvement resulting in readmission at the ED one week later. At this time, besides the symptoms previously described, he additionally complained of dyspnoea with minimal exertion and reported productive coughing and thoracalgia. He denied having experienced fever, night sweats, anorexia, abdominal pain, or vomiting.

On physical examination, he was apyretic and normotensive and had a normal and regular heart rate. He complained of shortness of breath and was found to be tachypnoeic, with a respiratory rate of 25 beats/minute and functional oxygen saturation (SaO_2_) of 89% on room air. Inspiratory crepitations and crackles in both lung bases were heard during chest auscultation. No palpable adenopathy and no other abnormalities were found during the examination.

Routine blood tests revealed normal neutrophil and eosinophil serum counts, with liver and renal function biomarkers also normal, blood glucose was 178mg/dL and C-reactive protein (CRP) was 10.7 mg/dL (normal: 0-0.5 mg/dL). Reverse transcriptase-polymerase chain reaction (RT-PCR) tests for influenza virus (A and B) and severe acute respiratory syndrome coronavirus 2 (SARS-CoV-2) were performed which came back negative, and the urinary antigen tests were also negative for *Streptococcus pneumoniae* and *Legionella pneumophila*.

**Figure 1:**
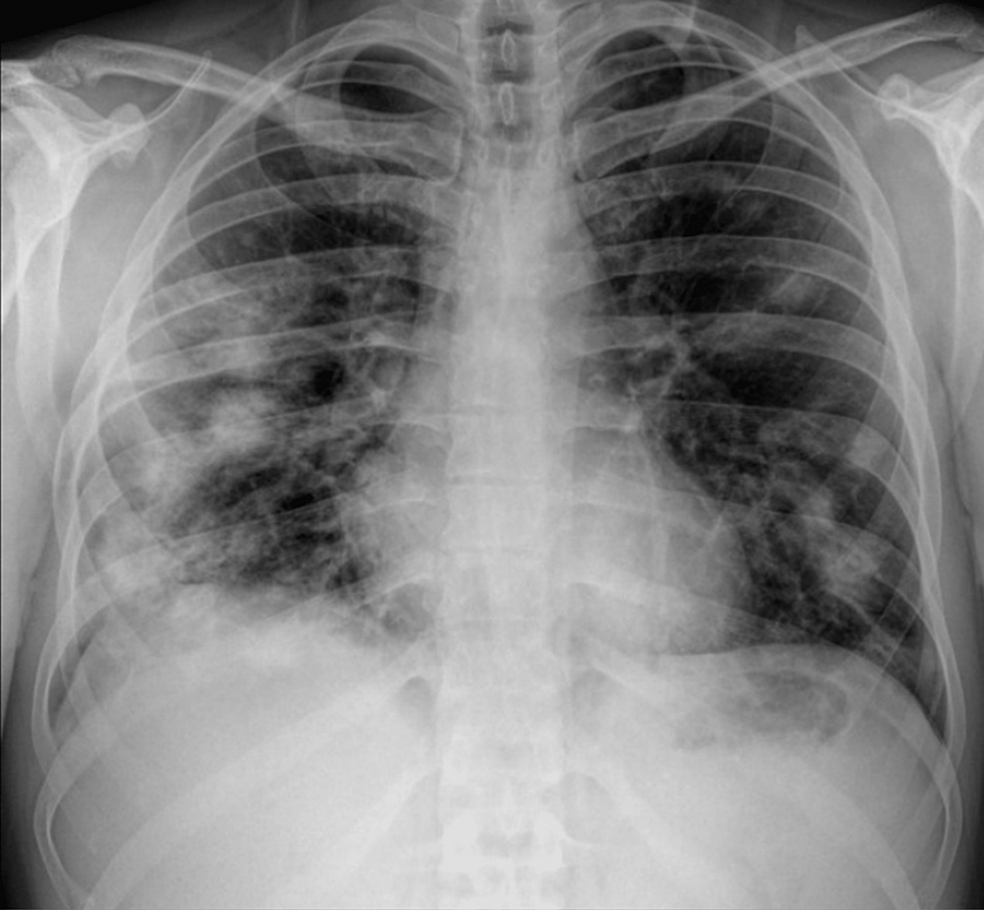
Chest X-ray (posterior-anterior view) upon admission to the ED.

An arterial blood gas test on room air revealed a low partial pressure of oxygen (PaO2) of 58 mmHg and a SaO_2_ of 90% without hyperlactatemia. A chest radiograph revealed the presence of diffused and irregular nodular parenchymal infiltrates in both lung fields, predominantly in the right lung (Figure 1).

**Figure 2:**
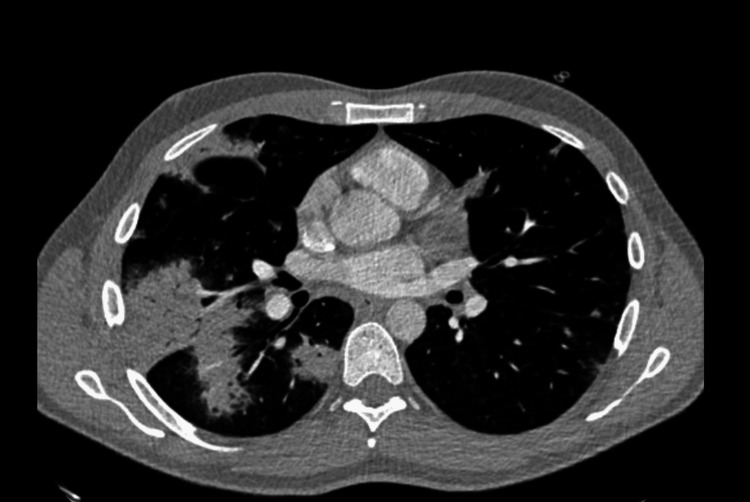
Chest CT scan performed in ED.

As a result of lung abnormalities seen on the chest X-ray, a computed tomography (CT) scan of the chest was performed (Figure 2), which showed the presence of a diffused interstitial pattern, ground-glass opacities, and bilateral consolidation mainly affecting the right lung, all of which were suggestive of an inflammatory or infectious lung parenchymal disease.

A preliminary diagnosis of community-acquired pneumonia was made by correlating the history, examination, and diagnostic tests. As per the institutional protocol, blood cultures were sent to a laboratory, and intravenous piperacillin-tazobactam as a first-line antibiotic therapy was started empirically. Subsequently, he was admitted to a hospital ward.

During hospitalisation, bronchoscopy with bronchoalveolar lavage (BAL) was conducted, but the results did not reveal any endobronchial lesions. BAL cell immunophenotyping showed an increase in lymphocytes (64%) but no alteration of the CD4+/CD8+ ratio. BAL cytology did not detect any malignant cells, nor did it reveal any viral, bacterial, fungal or mycobacterial pathogens.

Other diagnostic tests, such as serum protein electrophoresis, were consistent with an underlying inflammation or infection. However, there was no evidence of viral, bacterial, mycobacterial, or fungal infection present in tests conducted, and the blood cultures showed no growth of organisms. In addition, a comprehensive autoimmune study was negative.

**Figure 3:**
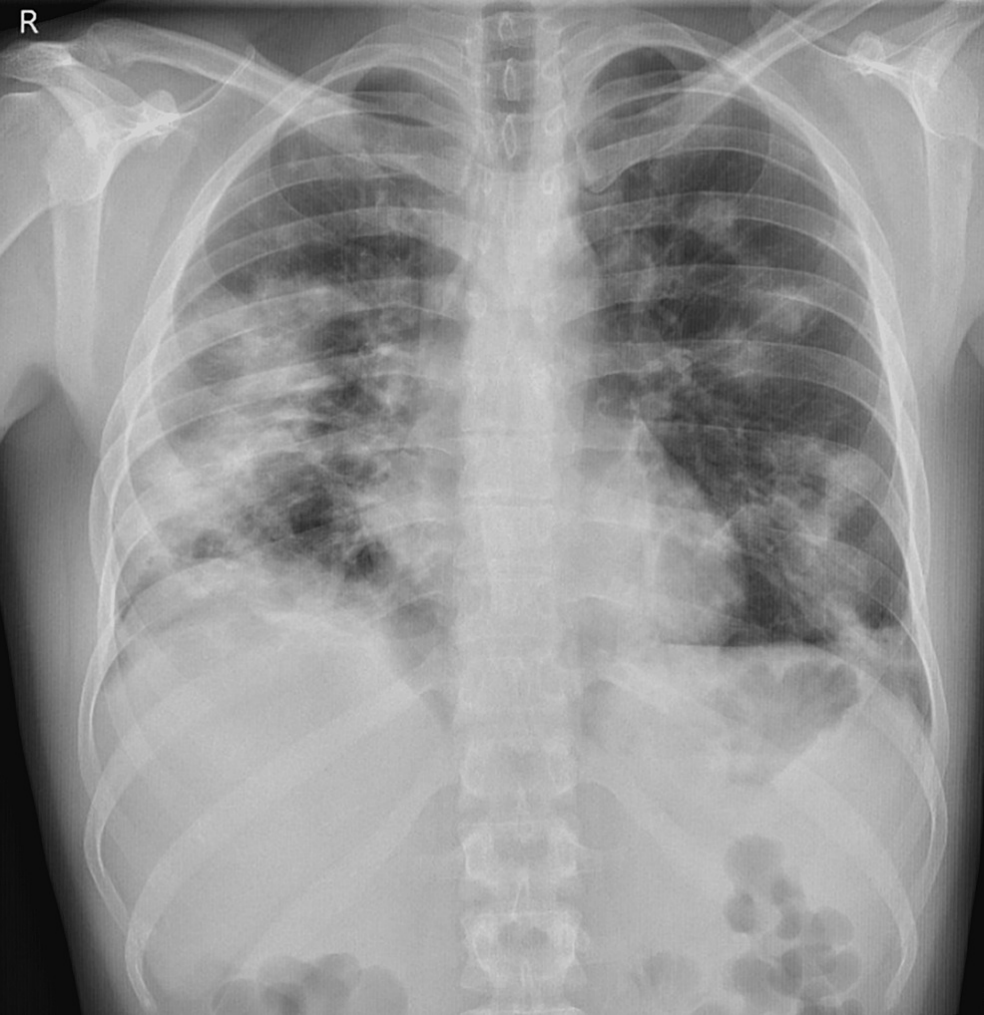
Worsening chest imaging (on the third day of admission).

Despite treatment, the patient's symptoms did not improve within 24-72 hours of commencing broad-spectrum antimicrobial agents, and there were no clinical, laboratory, or radiological improvements. The hypoxemia worsened, a fever developed, and a repeat chest X-ray on the third day showed worsening findings (Figure 3). Microbiological screening, including RT-PCR SARS-Cov2, was repeated, but the results remained negative.

Failure to respond to established antibiotic therapy raised the hypothesis of organising pneumonia (OP); however, it was difficult to distinguish whether the respiratory involvement was caused by an extraintestinal manifestation of UC or an adverse effect of SSZ therapy. Following a multidisciplinary discussion with radiology and pulmonology review, a decision to stop SSZ was made, and the use of corticosteroids with prednisolone (0.75mg/kg/day) was initiated. His insulin regimen was consequently adjusted.

**Figure 4:**
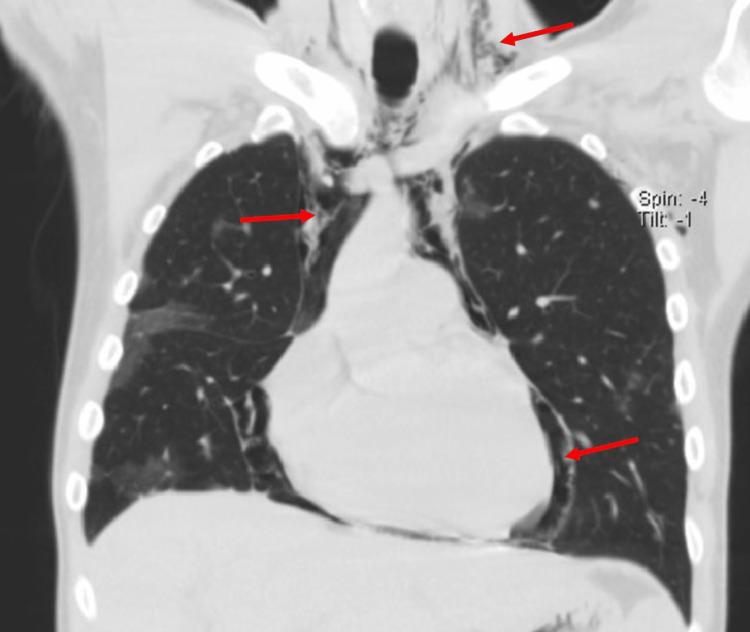
CT pulmonary angiogram (coronal view): red arrows indicate the presence of air leak syndrome.

A CT pulmonary angiogram (Figure 4) showed no features of pulmonary embolism; however, areas of lung parenchyma consolidation overlapping with those previously observed were detected, as well as pulmonary air leak syndrome, which included pneumomediastinum, pneumopericardium, pneumoperitoneum, pneumothorax, and subcutaneous emphysema.

**Figure 5:**
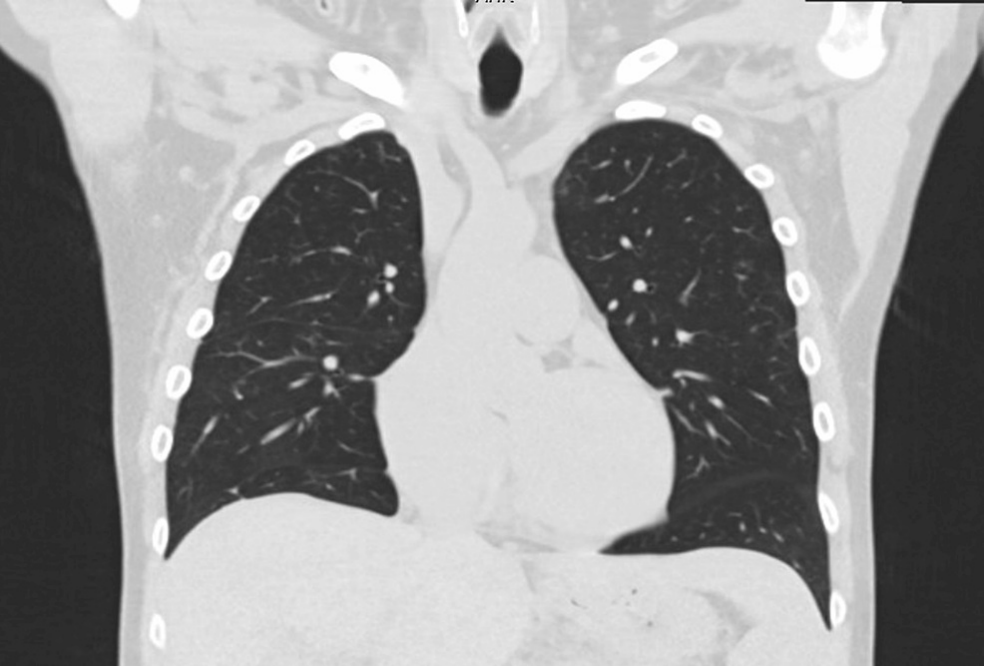
Chest CT scan (coronal view) reassessment six weeks after hospital discharge.

Although the patient was receiving corticosteroid medication, his condition worsened clinically, with a gradual worsening respiratory failure (fraction of inspired oxygen (FiO_2_) of 60%, arterial oxygen pressure (PaO_2_) of 57 mmHg on a venturi mask and a P/F ratio of 100). As a consequence, he required high-flow oxygen therapy and admission to the intensive care unit. After the corticosteroid dosage was increased to 80 mg of methylprednisolone (1.5 mg/kg/day), his condition gradually improved until he no longer needed oxygen therapy and acute care. His CRP levels significantly diminished to 1 mg/dL, and liver and kidney function biomarkers persisted unaltered. Consequently, prednisone was slowly tapered, and the patient was deemed medically fit for discharge on day 26. Six weeks post-discharge, the patient was seen in an outpatient follow-up appointment, and a subsequent chest CT revealed no areas of consolidation and a substantial improvement of the air leak syndrome findings (Figure 5).

## Discussion

Despite the fact that lung disease appears to be a rare extraintestinal manifestation of UC, a number of case reports have been published since the 1970s [5]. The pathophysiology of lung involvement in UC is still unclear; however, it could be explained by the fact that the colon and respiratory tract epithelium share a common embryologic origin and the same submucosal lymphoid tissue [3]. The severity of pulmonary manifestations can range from subclinical to more severe clinical forms. Subclinical forms are usually asymptomatic, not clinically evident, and only detected by imaging findings or pulmonary function tests. In severe forms, any part of the respiratory system can be affected, including the large airways, lung parenchyma, pleura, and pulmonary vasculature [2,5,6]. Involvement of the large airways is the most common, with bronchiectasis the most frequently reported form in 66% of cases [5]. Lung manifestations can occur in the early or late stages but do not always develop during the course of the disease. Furthermore, pulmonary findings may not coincide with periods of UC exacerbation and may even occur during periods of remission [4].

OP is a type of interstitial lung disease characterised by the presence of intra-alveolar granulation tissue [1]. In the absence of an etiological origin, it can be classified as cryptogenic OP. On the other hand, when attributed to specific causes, such as infections, autoimmune diseases, neoplasms, drugs, and radiotherapy, it is classified as secondary OP [1,3]. The symptoms and signs of OP are often indolent and nonspecific. The typical features of OP can vary among patients, ranging from mild symptoms, such as fever, cough, dyspnoea, and pleuritic chest pain, to more severe cases, such as severe respiratory failure and acute respiratory distress syndrome. Air leak syndrome, in which air or gases are present in extra-alveolar spaces and causing pneumomediastinum, pneumopericardium, pneumothorax, or subcutaneous emphysema can occur less frequently [5,7]. According to our research, Aydoğdu et al.’s study is the first case described in the literature of OP secondary to UC, which is complicated by air leak syndrome [7], and ours is the second.

The presence of multifocal patchy ground-glass opacities and consolidation on chest CT, along with the presence of lymphocytosis and a decreased CD4/CD8 ratio in the BAL, are features highly suggestive of OP [5,8]. However, histopathology is usually required to establish a firm diagnosis of OP since the clinical, laboratory, and radiological findings are often not specific [1]. In the absence of histopathological results, OP is usually diagnosed by ruling out other diseases and observing a response to corticosteroid treatment. In the present case, a transbronchial lung biopsy was not performed because the typical clinical and radiological features were suggestive of OP. Furthermore, the diagnostic work-up excluded other possible causes, such as malignancy, auto-immune conditions, and infectious causes.

Drug-induced OP was considered, namely associated with SSZ, but the inexistence of eosinophils in the BAL made it less likely. In many cases, it can be challenging to distinguish causality because OP can also be associated with an extraintestinal manifestation of UC [6,9]. Clinical and imaging improvement of OP following drug discontinuation is obviously the best clue to establish causality. However, there is no standard timing to attribute patient improvement to drug withdrawal.

Adequate organ support and corticosteroid therapy are the standard treatment of OP. Relapses have been reported upon stopping or reducing corticosteroids [3]. There are no clear guidelines on the ideal dosage and duration of corticosteroids; therefore, further research is necessary.

## Conclusions

OP is a lung condition that causes inflammation and scarring. Without treatment, it can cause serious lung damage, and in rare cases can be fatal if left untreated. Therefore, in patients with UC presenting with respiratory symptoms, it is crucial to consider OP as a differential diagnosis. Although rare, respiratory manifestations of UC can present in diverse forms which makes diagnosing a UC-associated lung disease challenging. In fact, its diagnosis is usually based on clinical and radiological criteria and occurs in the setting of a multidisciplinary team.

OP should be considered in all cases of persistent pneumonia that does not respond to antibiotic therapy and after other possible causes, such as malignancy and infection have been excluded. Although it is a histopathological diagnosis, OP is now a well-characterized entity with distinctive clinical and imaging features. Clinical confirmation of OP is often presumed when there is response to corticosteroid treatment. Due to its rarity, the authors of this article highlight the importance of including pulmonary manifestations of UC, especially OP, as prompt treatment with corticosteroids will promote recovery and prevent further complications.
